# Promoting adverse drug reaction reporting: comparison of different approaches

**DOI:** 10.1590/S1518-8787.2016050006122

**Published:** 2016-04-15

**Authors:** Inês Ribeiro-Vaz, Cristina Costa Santos, Ricardo Cruz-Correia

**Affiliations:** I Unidade de Farmacovigilância do Norte. Faculdade de Medicina. Universidade do Porto. Porto, Portugal; II Centro de Investigação em Tecnologias e Serviços de Saúde. Faculdade de Medicina. Universidade do Porto. Porto, Portugal; III Departamento de Ciências da Informação e da Decisão em Saúde. Faculdade de Medicina. Universidade do Porto. Porto, Portugal

**Keywords:** Drug-Related Side Effects and Adverse Reactions, Forms and Records Control, Drug Monitoring, Adverse Drug Reaction Reporting Systems, Pharmacovigilance

## Abstract

**OBJECTIVE:**

To describe different approaches to promote adverse drug reaction reporting among health care professionals, determining their cost-effectiveness.

**METHODS:**

We analyzed and compared several approaches taken by the Northern Pharmacovigilance Centre (Portugal) to promote adverse drug reaction reporting. Approaches were compared regarding the number and relevance of adverse drug reaction reports obtained and costs involved. Costs by report were estimated by adding the initial costs and the running costs of each intervention. These costs were divided by the number of reports obtained with each intervention, to assess its cost-effectiveness.

**RESULTS:**

All the approaches seem to have increased the number of adverse drug reaction reports. We noted the biggest increase with protocols (321 reports, costing 1.96 € each), followed by first educational approach (265 reports, 20.31 €/report) and by the hyperlink approach (136 reports, 15.59 €/report). Regarding the severity of adverse drug reactions, protocols were the most efficient approach, costing 2.29 €/report, followed by hyperlinks (30.28 €/report, having no running costs). Concerning unexpected adverse drug reactions, the best result was obtained with protocols (5.12 €/report), followed by first educational approach (38.79 €/report).

**CONCLUSIONS:**

We recommend implementing protocols in other pharmacovigilance centers. They seem to be the most efficient intervention, allowing receiving adverse drug reactions reports at lower costs. The increase applied not only to the total number of reports, but also to the severity, unexpectedness and high degree of causality attributed to the adverse drug reactions. Still, hyperlinks have the advantage of not involving running costs, showing the second best performance in cost per adverse drug reactions report.

## RESUMO

**OBJETIVO:**

Descrever diferentes abordagens de promoção da notificação de reações adversas a medicamentos entre os profissionais de saúde, determinando o seu custo-eficácia.

**MÉTODOS:**

Foram analisadas e comparadas estratégias adotadas pela Unidade de Farmacovigilância do Norte (Portugal) para promoção da notificação de reações adversas a medicamentos. As estratégias foram comparadas quanto ao número e relevância das notificações de reações adversas a medicamentos obtidas e quanto aos custos envolvidos. Os custos por notificação foram calculados somando os custos iniciais e os custos de manutenção de cada estratégia. Esses custos foram então divididos pelo número de notificações obtidas em cada intervenção, para avaliar o seu custo-eficácia.

**RESULTADOS:**

Todas as abordagens aumentaram o número de notificações de reações adversas a medicamentos. O maior aumento foi observado com os protocolos (321 notificações de reações adversas a medicamentos ganhas, custando 1,96 € cada), seguidos pela primeira abordagem educacional (265 notificações, 20,31 € cada) e pela colocação de *hyperlinks* (136 notificações, 15,59 € cada). Com relação à gravidade das reações adversas a medicamentos, os protocolos foram a estratégia mais eficiente, custando 2,29 € cada notificação, seguida da colocação de *hyperlinks* (30,28 € cada, sem custos de manutenção). Quanto às reações adversas a medicamentos inesperadas, o melhor resultado pertenceu aos protocolos (5,12 € cada notificação), seguido por uma primeira abordagem educativa (38,79 € cada notificação).

**CONCLUSÕES:**

Os autores recomendam a implementação de protocolos em outros centros de farmacovigilância. De fato, estes parecem ser a intervenção mais eficaz, permitindo receber notificações de RAM com custos mais baixos, aplicando-se este aumento tanto ao número total de notificações de reações adversas a medicamentos, como à gravidade, imprevisibilidade e alto grau de causalidade atribuído a elas. Ainda assim, a colocação de *hyperlinks* apresenta a vantagem de não envolver custos de manutenção, por isso tem o segundo melhor desempenho no indicador custo por notificação de reações adversas a medicamentos.

## INTRODUCTION

Adverse drug reactions (ADR) are inherent to medicine use^20^, and most of them can only be detected after the commercialization of the drug^14^. In fact, during clinical trials, rare reactions are hardly detected, as well as the ones associated with chronic utilization of the drug. It is also difficult to predict the drug effect among special populations (pregnant women, children, older adults), as they usually do not participate in the clinical research.

Because of these limitations, post-marketing surveillance is essential, which is why most countries have pharmacovigilance centers to monitor detected ADR. The fundamental tool used by these centers is the spontaneous reporting of ADR by healthcare professionals and consumers. This method consists in describing an adverse episode suspected to be caused by one or more drugs and provides valuable information to the regulatory health authorities, which is important for the decisions about marketed medicines. The biggest problem of this method is the underreporting, i.e., ADR are detected but not reported to national regulatory health authorities. Most developed countries face this situation^15^ ^,^ ^19^. Worldwide, many approaches have been completed to fight the major problem of ADR underreporting, such as regular visits to health professionals^10^, questionnaire studies^2^, educational interventions (including workshops, meetings and presentations)^4^ ^,^ ^12^ ^,^ ^16^, among others.

This study aimed to describe several approaches that intended to improve ADR reporting and determine the cost-effectiveness of each one of them.

## METHODS

From its creation (in 2000) to 2003, Northern Pharmacovigilance Centre, a Portuguese regional pharmacovigilance center, had an extremely low rate of ADR reports, about 43 per year/million inhabitants. We realize this value is very low when compared with the World Health Organization (WHO) recommendation for an Optimal National Centre, which is at least 200 reports per year/million inhabitants^a^.

To reach its objectives, in 2004 the Centre established a collaboration protocol (*protocol approach*) with the immunoallergology department of a central hospital (located on the same street as the Centre) to collect every suspected case of ADR emerged in appointments related to drug allergies. This collaboration includes regular visits of the pharmacovigilance staff to the immunoallergology department to collect the detected cases in ADR report forms, under the physician supervision. Then, the form is signed by the physician and follows the normal course of all the ADR spontaneous reports. This approach was replicated two more times, in 2007 and 2009, in two other immunoallergology departments, one from a specialized hospital (pediatric hospital, located 6 km from the Centre) and another from a central hospital (located 11 km from the Centre). These three protocols remain active.

A study conducted in 2004 provided educational interventions (*educational approach*) for physicians and pharmacists^4^ ^,^ ^8^. Those interventions were based on a previous case-control study that identified the reasons for underreporting^6^ ^,^ ^7^. The educational approach includes workshops about pharmacovigilance at health care professionals’ working places.

Since the effect of educational interventions decreased over time, the authors of the previously described work promote reinforcement interventions (*educational and telephone approach*). We started a new study in 2007, also among physicians and pharmacists. This study consisted not only in outreach interventions (workshops), but also in telephone interviews^9^ ^,^ ^17^. The phone interviews followed a script about ADR and the importance of reporting. Details are described in a previous publication^17^.

**Figure 1:**
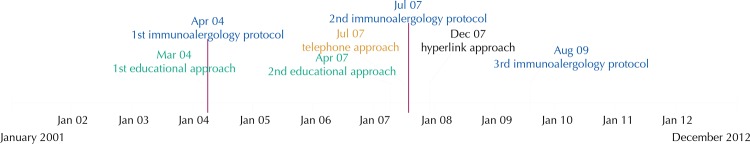
Timeline of the studied approaches.

We propose a new approach: the inclusion of a hyperlink (*hyperlink approach*) to an online ADR reporting form on hospitals’ electronic patient records (EPR). The main aim of this study, performed from 2006 to 2010, was to evaluate the impact of these hyperlinks on the number of spontaneous ADR reports^18^. The inclusion of hyperlinks began in December 2007 and continued over the following five months. The temporal distribution of all these approaches is shown in Figure 1.

In the present work, we analyzed the number of ADR reports obtained with each one of the described approaches. We know exactly which ADR reports were originated at the three departments participating in the protocol intervention and analyzed them separately. Four physicians were involved.

The first educational intervention (in 2004) involved three hospitals, 26 healthcare centers, and 73 pharmacies. About 900 health care professionals attended these interventions^4^. About 340 health care professionals (physicians and pharmacists) attended the second intervention (second workshop + telephone, both in 2007). Five health care centers, two hospitals, and 40 pharmacies received the telephone intervention, and 16 health care centers, two hospitals, and 23 pharmacies received the second educational intervention.

For the hyperlinks, we estimated 15,000 health care professionals potentially affected by the intervention, as this is the total number of professionals working at the 12 participating hospital centers (corresponding to 22 hospitals). It was the first exposure to any intervention for eight of these hospital centers.

The variables analyzed were: type of approach, ADR relevance, initial costs of the interventions, running costs of the interventions, and costs per ADR report. Each of these variables is described as follows.

- Type of approach: hyperlink, protocol, educational, and telephone approach.
- Number of ADR reports obtained with each intervention: the difference between ADR reports received two years after the intervention and ADR reports received two years before the intervention.
- ADR relevance: we adopted the following criteria: (1) ADR severity; (2) ADR expectedness; and (3) causality attributed to the ADR report. A serious ADR is any untoward medical occurrence that results in death, requires inpatient hospitalization or prolongation of existing hospitalization, results in persistent or significant disability or incapacity, or is life-threatening^2^. An unexpected ADR is the one in which the nature or severity is not consistent with domestic labeling or market authorization, or expected from characteristics of the drug^2^. We considered an ADR more relevant if one of the two highest degrees of causality was attributed to it: (1) definitive or certain, or (2) probable (the medicine was the likely causative agent of an observed adverse reaction)^b^.
- Initial costs of the interventions: we consider as initial costs the expenses needed for implementing the approach, as educational material and staff working hours. These costs are described in Table 1.
  **Table 1:** Estimated costs of each approach.

Approach	Initial costs		Annual running costs	
	
	Value	Description	Value	Description
Protocol approach	150 €	Pharmacovigilance and clinical service staff working hours	240 €	Fuel, material, and pharmacovigilance and clinical staff working hours
Hyperlink approach	2,120 €	Pharmacovigilance and software development staff working hours	-	-
Educational approach	200 €	Educational material and pharmacovigilance staff working hours	2,500 €	Fuel, material, and pharmacovigilance staff working hours
Telephone approach	400 €	Telephone calls during the pilot study and pharmacovigilance staff working hours	800 €	Telephone calls, material, and pharmacovigilance staff working hours
- Running costs of the interventions: annual running costs are the expenses needed for continuing the projects, as fuel, material and staff working hours. These costs are described in Table 1. We did not consider the normal (daily) costs of ADR report processing, as we only meant to compare the costs involved in obtaining ADR reports.
- Costs per ADR report: costs by ADR report were estimated by adding initial costs and running costs. Initial costs per ADR report were obtained by dividing initial costs by the difference between ADR reports received two years after the intervention and ADR reports received two years before the intervention (which we consider to be the number of notifications obtained with each intervention). Running costs were obtained by dividing the running costs of the two-year intervention by the number of notifications obtained with each intervention. To assess the cost-effectiveness of each intervention, we considered the sum of these costs (initial + running costs) as the total cost of each ADR obtained in the two years following each intervention.

The pharmacovigilance center website uses a web server and has audit trails that read each site visit since 2006. These audit trails are processed using the Webalizer program (www.webalizer.org) to estimate site hits, user logins and visits. ADR reports obtained by these approaches are included in a database. We collect them by selecting the report date and origin.

We presented the total number of reports received in each quarter during the period studied. For each health institution, ADR reports made before and after the intervention, if any, were measured.

To examine whether each intervention increased the ADR report trend, an interrupted time series analysis using autoregressive integrated moving average (ARIMA) was performed using quarter data of ADR reports, as well as each intervention (first and second educational approach, telephone approach, and hyperlink approach) as dichotomous variables (before and after intervention).

We performed an additional analysis with the hyperlinks approach, to consider the institutions exposed to any type of intervention for the first time. With this sub-analysis we intended to isolate the ADR reports obtained with each intervention.

This study was approved by the local Ethics Committee of the Faculdade de Medicina of the Universidade do Porto (Process PCEDCSS-FMUP 08/2014, approved in May 7, 2014).

## RESULTS

**Figure 2:**
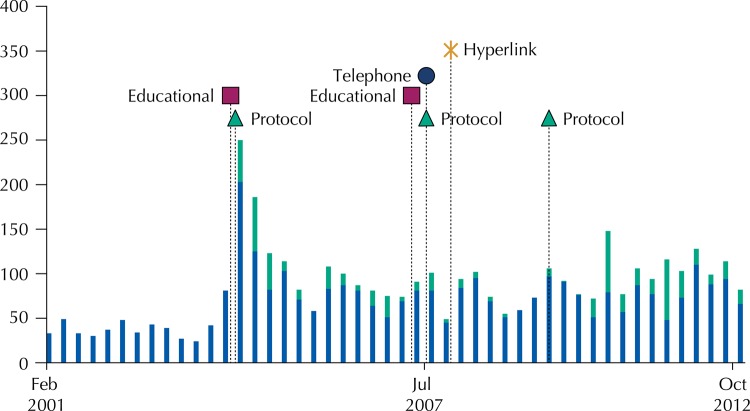
Total number of adverse drug reaction reports received in the Northern Pharmacovigilance Centre during the studied period, per trimester (in green, those obtained with protocols).

We found an increasing trend in the number of ADR reports received by the Northern Pharmacovigilance Centre during the studied period: 2000-2012. The number of annual ADR reports increased from the year in which the first interventions were made (2004) to the end of the study period (Figure 2).

Excluding the ADR reports obtained with the protocol approach, the only intervention that significantly increased the ADR report trend was the first educational approach, in the first quarter of 2004 (p < 0.001).

We did not find a significant increase in the ADR report trend in the second educational approach in second quarter of 2007 (p = 0.203). The telephone approach also failed to significantly increase ADR reporting in the third quarter of 2007 (p = 0.243). With the hyperlink approach we observed a slight increase in ADR reporting, although without statistical significance (p = 0.193).

All the approaches increased the number of ADR reports, when we compare the two years before with the two years after the interventions. We noted the biggest increase with the protocol approach (321 ADR reports obtained), followed by the first educational approach, with 265 ADR reports obtained, and by the hyperlink approach, with 136 ADR reports. For the hyperlink approach, we isolated the institutions exposed to an intervention for the first time; these cases obtained 141 ADR reports.

According to the initial costs involved, our results suggest that the protocol approach is the most cost-effective, costing 0.47 € per ADR report, followed by the first educational and telephone approach, costing 0.78 € per ADR report. Analyzing running costs, the hyperlinks approach is the most favorable, having none. On the other hand, we can conclude that the second educational approach is the intervention that entails more costs, with 123.81 € per report (Table 2).

**Table 2:** Number and costs of adverse drug reaction reports obtained with each intervention.

Approach	Intervention	Adverse drug reaction reports				Costs (€) per report		

		Before		After				
		
		2 years	1 year	1 year	2 years	Initial costs	Running costs (2 years)	Total
Protocols		0	0	204	117	0.47	1.49	1.96
Hyperlinks		153	120	277	132	15.59	0.00	15.59
Hyperlinks NPE*		68	47	146	110	15.03	0.00	15.03
Educational	1^st^ workshop	36	24	257	68	0.78	19.53	20.31
	Pharmacies	2	8	110	25	1.60	40.00	41.60
	Health care centers	25	7	102	17	2.29	57.47	59.77
	Hospitals	9	9	45	26	3.77	94.34	98.11
Phone Interview		47	26	87	34	8.33	33.33	41.67
	Pharmacies	23	10	37	14	22.22	88.88	111.11
	Health care centers	3	1	8	2	66.67	266.67	333.33
	Hospitals	21	15	42	18	16.67	66.67	83.33
Educational	2^nd^ workshop	54	40	106	30	4.76	119.05	123.81
	Pharmacies	39	25	69	18	8.70	217.39	226.09
	Health care centers	10	13	26	6	22.22	555.55	577.78
	Hospitals	5	2	11	6	20.00	500.00	520.00

* NPE: Not previously exposed. Considering only the institutions without any previous intervention.

Regarding the relevance of ADR reports, we analyzed the severity, expectedness and degree of causality attributed to the reports. Regarding serious ADR, the protocol approach was the most cost-effective, costing 2.29 € per report. The hyperlink approach obtained the second lowest value (30.28 € per report), having no running costs. We found similar results for the relevance criterion of causality assessment. Concerning ADR expectedness, the best result belonged to the protocol approach (5.12 € per report), followed by the first educational approach (38.79 € per report) (Table 3).

**Table 3:** Number and costs of serious, unexpected, and classified with a high degree of causality adverse drug reaction reports obtained with each intervention.

Approach	Intervention	Adverse drug reaction reports				Costs (€) per report		

		Before		After				
		
		2 years	1 year	1 year	2 years	Initial costs	Running costs (2 years)	Total
Serious								
Protocols		0	0	180	94	0.54	1.75	2.29
Hyperlinks		113	96	193	86	30.28	0.00	30.28
Educational	1^st^ workshop	12	15	111	42	1.59	32.68	34.27
Phone interview		29	19	55	21	14.29	57.14	71.43
Educational	2^nd^ workshop	37	23	44	8	-	-	-
High degree of causality								
Protocols		0	0	165	66	0.65	2.08	2.73
Hyperlinks		114	86	232	109	15.03	0.00	15.03
Educational	1^st^ workshop	14	17	169	47	1.08	27.03	28.11
Phone interview		40	17	65	26	11.76	47.06	58.82
Educational	2^nd^ workshop	37	27	74	22	6.25	156.25	162.50
Unexpected								
Protocols		0	0	70	53	1.22	3.90	5.12
Hyperlinks		63	40	69	37	706.67	0.00	706.67
Educational	1^st^ workshop	10	7	111	40	1.49	37.30	38.79
Phone interview		17	11	22	7	400.00	1,600.00	2,000.00
Educational	2^nd^ workshop	24	17	28	6	-	-	-

## DISCUSSION

Although there is some overlap of interventions, making it difficult sometimes to differentiate the gains from each one of them, our results show that, in general, all interventions increased the number of ADR reports when comparing two years before with two years after.

Protocols in hospital immunoallergology departments seem to be the most efficient intervention. In fact, this intervention is the one that allows obtaining ADR reports with lower costs involved, with an increase not only in the total number of ADR reports, but also in the severity, unexpectedness, and high degree of causality attributed to the ADR.

Nevertheless, these protocols have the disadvantage of increasing the reports of ADR in patients of a specific population (patients with allergies), which can bias the global pharmacovigilance data. We started to establish these protocols at the request of one of the immunoallergology departments, but we are trying to establish similar protocols in other departments (as oncology departments, hospital pharmacies, among others), to solve the bias issue.

On the other hand, the hyperlink approach has the great advantage of not involving running costs, and seems to have the second best performance in costs per ADR report. Even when we consider only the hospitals exposed to an intervention for the first time (to avoid the overlap effect), this behavior remains.

We also concluded that the first educational intervention was much more efficient than the second one. In fact, the second intervention seemed to be counterproductive, as shown by the results of serious and unexpected ADR reports (these numbers decreased after the intervention). We already had this conviction since this intervention was performed. In fact, in most health care institutions where the second intervention took place, we found professionals less receptive than in the first intervention, as they already knew the subject and did not seem to believe they needed another workshop about it.

Unfortunately, we are not able to compare our results with other authors’ results, as we failed to find any study addressing the issue of ADR report costs. Many studies proposed strategies to improve ADR reports^1^ ^,^ ^11^ ^,^ ^13^ and some authors have already studied the costs of an ADR^3^ ^,^ ^5^. However, no one had studied the costs involved in obtaining ADR reports before, which is the novelty of our work.

Although there might be some overlap and eventual contamination among the interventions, we believe that this did not introduce an important bias in our conclusions. First, we knew exactly which reports were originated at the departments participating in the protocols. Moreover, we included in our results the ADR reports obtained after the hyperlink inclusion in the hospitals that had an intervention for the first time. Thus, we could infer that the gain in ADR reports after hyperlink inclusion was caused by this intervention. Furthermore, there is no problem of overlapping for the first educational approach (workshops in 2004) because this was the first intervention made. The only interventions for which we cannot resolve the overlapping limitation is the second educational intervention and the phone intervention. However, these two interventions were planned as complementary to the first one.

We believe that our work can help pharmacovigilance centers worldwide choose the best set of interventions to promote adverse drug reactions report. This choice must be based on the particular characteristics of each center, such as available staff and budget, geographic location, proximity to hospitals, among others.

Based on our results, we recommend the implementation of protocols with hospital immunoallergology departments, as they seem to be the most cost-effective intervention, followed by hyperlinks to ADR reporting forms, and the promotion of educational interventions to health care professionals for the first time.

## Footnotes

a Governo de Portugal, INFARMED – Autoridade Nacional do Medicamento e Produtos de Saúde. *Farmacovigilância em Portugal*. Lisboa: INFARMED; 2004.

b World Health Organization; Uppsala Monitoring Centre Uppsala. Uppsala: Uppsala Monitoring Centre Uppsala [updated 2015 Aug 18; cited 2013 Aug 9]. Available from: http://www.who-umc.org/DynPage.aspx?id=22682
